# Efficacy of Levobupivacaine Versus Levobupivacaine Plus Dexmedetomidine Infiltration for Post-Tonsillectomy Analgesia: A Randomized Controlled Trial

**DOI:** 10.1155/2022/9958668

**Published:** 2022-09-19

**Authors:** Ghada Mohammad Abo Elfadl, Marwa Mahmoud AbdelRady, Hany M. Osman, Mohamed Omar Gad, Nessren M. Abd el-Rady, Wesam Nashat Ali

**Affiliations:** ^1^Anesthesia and Intensive Care Department, Faculty of Medicine, Assiut University, Assiut, Egypt; ^2^Lecturer of Otorhinolaryngology Head and Neck Surgery, Faculty of Medicine, Assiut University, Assiut, Egypt; ^3^Assistant Professor of Medical Physiology Department, Faculty of Medicine, Assiut University, Assiut, Egypt; ^4^Medical Physiology Department, Sphinx University, New Assiut, Assiut, Egypt

## Abstract

**Background:**

The study evaluated the analgesic effects of levobupivacaine infiltration in the tonsil bed, and a combination of levobupivacaine and dexmedetomidine in patients undergoing tonsillectomy.

**Methods:**

Ninety children (ages 3 to 7 years) who were scheduled for a tonsillectomy were allocated randomly into two groups. (L Group**)**: peritonsillar infiltration with 0.25% levobupivacaine (2 ml + 0.5 ml saline 0.9% per tonsil). (LD Group): levobupivacaine 0.25% (2 ml) plus dexmedetomidine 1 *μ*g/kg diluted in 1 ml saline 0.9% (0.5 ml in each tonsil), and administered by peritonsillar infiltration (2.5 ml per tonsil) following intubation 3–5 minutes before operation. To avoid bias, infiltrate a total volume of 2.5 ml in each tonsil. The first analgesic request time was the primary outcome, with postoperative pain score, total analgesic consumption, total oral intake, sedation, and side effects as secondary outcomes.

**Results:**

The first rescue analgesia time in the LD group was longer (644.31 ± 112.89 min) than in the *L* group (551.51 ± 146.16 min, *P*-value <0.001). The number of patients who required >1 analgesic dose in the *L* group (*n* = 13) was higher than in the LD group (*n* = 5). The LD group consumes a lower total dose of IV paracetamol in the first 24 hours postoperatively (321.89 ± 93.25 mg) than the *L* group (394.89 ± 183.71 mg, *P* < 0.00-value < 0.050). On the first day postoperatively, patients in the LD group had a higher total oral intake (*P* < 0.001). Except for a slight increase in laryngospasm in the *L* group, there were no side effects.

**Conclusions:**

The Children's peritonsillar infiltration of levobupivacaine and dexmedetomidine improved postoperative pain after adenotonsillectomy. The topically applied levobupivacaine and dexmedetomidine were concomitant with no systemic effects, greater total oral intake on the first day postoperative, and higher family satisfaction.

## 1. Introduction

Tonsillectomy is one of the most popular surgical interventions in children [1]. Patients commonly experience pain with swallowing after this treatment [2]. Sore throats postoperatively are a serious issue since they can cause decreased oral intake and dehydration, both of which are hazardous [3].

Late postoperative risks from a sore throat include epithelial loss in the surgical site and necrosis in soft tissue, bleeding in dehydrated persons, acute pain, and a delay in recovery. Several tonsillectomy studies have demonstrated that a local anesthetic (LA) injection that is, routinely given before procedures to reduce pain stimulation during the procedure is effective [4, 5].

Various treatments have been attempted with varied results to minimize postoperative discomfort and boost patient and parent satisfaction following adenotonsillectomy. As a result, peritonsillar local anesthetic infiltrations, especially long-acting bupivacaine, are also employed [4].

The ability of local anesthetic is to not only block the transmission of peripheral pain receptors following tissue damage but also to avoid central nervous system sensitization is why it is used in the perioperative stage [6].

Levobupivacaine is an amide-type long-acting local anesthetic that is gentler on the cardiac and nervous systems [7].

Dexmedetomidine (DEX) is utilized in pediatric patients for analgesia and sedation, as well as in the intensive care unit and during noninvasive (MRI) and invasive (endoscopy and cardiac catheterization) procedures [8]. It can also reduce the use of opioids and anesthetic requests, as well as decrease developing delirium [9] and postanesthesia shivering, according to adult studies [9–11].

The objective of this study was to compare the analgesic effects of levobupivacaine infiltration in the tonsil bed with a combination of levobupivacaine and dexmedetomidine in patients undergoing tonsillectomy.

## 2. Materials and Methods

The Medical Research Ethics Committee of the Faculty of Medicine provided ethical permission for this randomized, prospective, double-blind comparative study on September 29, 2019. (Approval no: 17300316). It was registered on ClinicalTrials.gov (NCT04113720) and tracked the Helsinki Declaration guidelines. All the patients' guardians gave written informed consent when the study's purpose was outlined.

The study enrolled 90 patients (ages 3 to 7 years) who were designated by the American Society of Anesthesiologists (ASA) I-II for elective tonsillectomy with or without adenoidectomy (possibly via surgical retraction and bipolar diathermy).

Previous peritonsillar abscess, obstructive sleep apnea syndrome (whether or not established by a polysomnography test), cardiovascular, liver, or kidney disease, unsatisfactory preoperative peripheral arterial oxygen saturation, coagulation disorders, relevant drug allergies, neurological or psychiatric illness, patients who take analgesics regularly or who have taken analgesics 24 hours before surgery, and finally, patients who have difficulty perceiving anesthesia.

Randomization and blinding: randomization occurred the morning of the procedure before general anesthesia was administered.

Ninety patients were randomly assigned into two groups (*n* = 45) via a computer-generated randomization technique.

The first group (Group L) received levobupivacaine 0.25% via peritonsillar infiltration (2 ml + 0.5 ml saline 0.9% per tonsil) following intubation 3–5 minutes before operation.

The second group (Group LD) received levobupivacaine 0.25% (2 ml) plus dexmedetomidine 1 *μ*g/kg diluted in 1 ml saline 0.9% (0.5 ml in each tonsil) and administered by peritonsillar infiltration (2.5 ml per tonsil) after intubation 3–5 minutes before the beginning of the operation. To avoid bias, infiltrate a total volume of 2.5 ml in each tonsil.

The selected dose of dexmedetomidine (1 *μ*g/kg) was consistent with prior studies showing the analgesic efficiency of dexmedetomidine at 1 *μ*g/kg instead of 0.5 *μ*g/kg and 0.75 *μ*g/kg confirmed [12].

The research medicines were prepared in an identical syringe by a nurse who was not participating in the study. All of the syringes had numbers ranging from 1 to 90 and these numbers were saved in opaque envelopes. Only one anesthesiologist who packed the envelopes had access to the codes on the envelopes. All study personnel, including patients' guardians, were unaware of the treatment assignment.

### 2.1. Anaesthetic Technique

All children were required to fast for at least 6 hours before surgery, with clear fluids permitted until 2 hours before anesthetic induction. Blood pressure, heart rate, oxygen saturation, and end-tidal CO_2_ were assessed in the operating room. The anesthetic protocol was fixed. All subjects were preoxygenated with 100% oxygen for 3 minutes using a facemask. To produce anesthesia, incremental 1.5% sevoflurane dosages up to 7% were utilized in a 70% oxygen/air mixture. Dexamethasone (0.2 mg/kg, maximum dose of 8 mg), and an intravenous antibiotic were given. There were no NSAIDs, opioids, or paracetamol used throughout the procedure.

After achieving neuromuscular block with cis-atracurium 0.3 mg/kg, the endotracheal tube was introduced, and anesthesia was continued with sevoflurane at 2.5% in a 70% oxygen/air mixture. Before the surgery, the study medicines were injected pericapsularly via the tonsil bed and peritonsillar tissue in a fan-shaped pattern from the top to the lower pole of the tonsil fossa via a syringe with a 25-gauge spinal needle. When the anesthetic gases were switched off at the finale of the surgery, the neuromuscular blockade was countered with 0.05 mg/kg neostigmine and 0.02 mg/kg atropine, and the patients were turned away in the recovery position. The children were extubated awake and transported to the postanesthesia care unit (PACU) after the protective airway reflexes were confirmed to have returned. Supplemental oxygen was withheld if the child could maintain a SaO_2_ >95% in ambient air for 5 minutes. After earning an Aldrete score of 9 or higher, participants were discharged from PACU to the ward [13].

### 2.2. Assessment Parameters

(i) The patient's demographic and clinical data are age, sex, weight, height, and ASA class.
(ii) Operative room data include;
  - Vital signs, such as noninvasive arterial blood pressure, heart rate, and peripheral arterial oxygen saturation were continuously monitored and noted before, during, and after the administration of study drugs, as well as at 15, 20, 25, and 30 minutes during operation.
  - Time of anesthesia (from initiation of anesthesia till extubation).
  - Operation time (from the beginning of the operation to the end of the bleeding control).
  - Time to extubation (from the cessation of anesthesia to extubation).
(iii) PACU and ward data include;
  - Hemodynamic parameters: heart rate, mean arterial blood pressure, and peripheral arterial oxygen saturation were measured and noted in the PACU (time of PACU arrival is 0 min) and at 15, 30, 45, and 60 min postoperative.
  - Pain assessment: via the Children's Hospital of Eastern Ontario Pain Scale (CHEOPS) [14], on arrival to PACU at 0, 30, 60, 90 min, 2, 6, 10, 12, and 24 h after recovery from anesthesia. IV paracetamol 15 mg/kg was given for rescue analgesia if two double notes separated by a 5 min waiting period produced CHEOPS >6.
  - Ramsay sedation scale: [15] noted in 0 (upon arrival at the PACU), 15, 30, 60, 120, 180 and 240 minutes postoperatively.
  - The time to first request analgesia and total analgesic intake in the first 24 hours postoperatively were recorded.
  - The number of rescue analgesic dosages consumed following surgery.
  - For the first 24 hours after surgery, the total oral intake (fluids and semisolids).
  - Perioperative side effects: were recorded and treated (such as hypotension, hypertension, tachycardia, bradycardia, hypoxia, arrhythmia, excessive secretions, bleeding, respiratory depression, nausea, and vomiting).
  - The participant's parents rated their satisfaction with the analgesia at the final of the 24-hour study period via a five-point Likert scale (1 - very dissatisfied, 2 - dissatisfied, 3 - neutral, 4 - satisfied, and 5 - very satisfied).

All parents received a call from the same convalescent nurse the day after surgery, asking if they had seen any after effects. The patients were then observed for a week to see if any problems emerged. Upon discharge, all children were given oral paracetamol (20 mg/kg) as needed (a maximum of four times in 24 hours).

### 2.3. Surgical Work

Complete bed dissection was conducted using cold instruments in all patients, with no radiofrequency, diathermy, or LASER usage. In all cases, the lower pole was ligatured (with 2–0 silk), and hemostasis was obtained with bipolar cautery. The surgeon used sharp adenoid curettes to do the adenoidectomy (if necessary), and he palpated the adenoid bed. The curettage was repeated if necessary to ensure complete eradication [16]. A single otolaryngologist surgeon did all procedures.

### 2.4. Outcomes

The assessment of the first analgesia rescue call was the primary outcome. Secondary outcomes involved the effect of peritonsillar infiltration on postoperative recovery in children undergoing tonsillectomy and adenoidectomy, such as pain scores, total analgesic consumption, hemodynamics, total oral intake, and sedation, as well as recording any adverse effects over the 24-hour trial period.

### 2.5. Statistical Analysis

Power of the study: the trial's primary outcome was the period of postoperative analgesia as measured by the first call for analgesics. A target sample size was determined based on a pilot study's findings. According to a power analysis, a sample size of 41 patients in each group would have 95% power to detect a difference of 0.8 effect size in the time to the first request for rescue analgesics between the two groups at the 0.05 level of significance. To account for patient dropout, a total of ninety individuals were registered.

### 2.6. Data Analysis

The Shapiro–Wilk test was performed to define the baseline variable distribution. To examine continuous variables reported as mean, the Student's *t*-test and one-way analysis of variance (ANOVA) test with posthoc multiple comparisons were utilized (SD). The nonparametric data from the two groups, reported as medians, were compared by the Mann–Whitney *U* test (range). Categorical data, described as numbers and percentages, were examined with the Chi-square or Fisher exact test. A statistically significant *P* value of 0.05 was used. IBM SPSS Statistics Version 20 (SPSS Inc, Chicago, IL, USA) was utilized for all statistical studies.

## 3. Results

**Figure 1:**
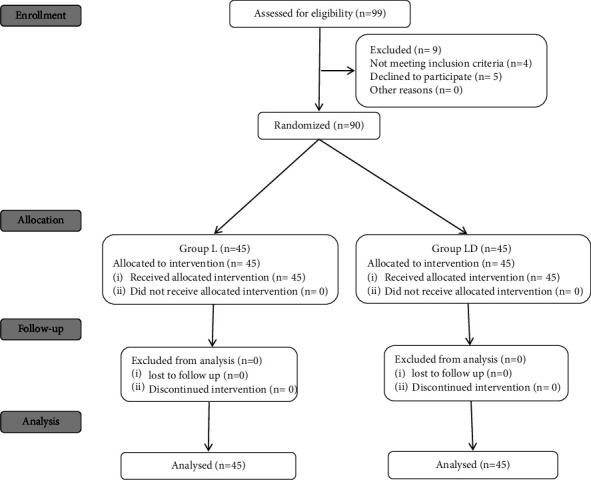
The consort flow chart.

Amongst 99 participants who were screened for eligibility, ninety patients were recruited for the study, each group contained forty-five patients (Figure 1).

There were no significant differences among the groups of participants regarding age, weight, height, gender, time of operation, and anesthesia (Table 1).

### 3.1. The Rescue Analgesia and Analgesic Consumption

CHEOPS score was higher in the (*L* group) requiring rescue analgesia at (551.51 ± 146.16 min), whereas in the (LD group), the CHEOPS score started to increase and required rescue analgesia at (644.31 ± 112.89 min).

Not only was the time to the first rescue analgesic dose significantly shorter in the (*L* group) (*P* value 0.001), but the number of patients who required more than one rescue analgesic dose was also higher in the (*L* group) (*n* = 13, 28.9%) than in the (LD group) (*n* = 5, 11.1%) (*P* value 0.050).

Over 24 hours, postoperative rescue analgesia was provided with IV paracetamol bolus 15 mg/kg as needed or if the CHEOPS score was >6. When compared to the *L* group, the mean total paracetamol dose of rescue analgesia taken in the first 24 hours postoperatively was considerably lesser in the LD group (321.89 ± 93.25 mg) than in the *L* group (394.89 ± 183.71 mg, *P* < 0.001 value < 0.050) (Table 2).

The extubation time was significantly longer in (the LD group) (6.2 ± 0.7 min) when compared with (the *L* group) (5.1 ± 0.8 min, *P* < 0.001), leading to slight prolongation in anesthesia times in (the LD group) (42.8 ± 3.8 min), more than that in (the *L* group) (41.8 ± 3.1 min) but with no significant difference (Table 1).

The maximum mean values for total oral dose were achieved for 24 hours for liquids and semisolids in the LD group (725.33 ± 95.12 ml and 630.9 ± 139.39 ml, *P* < 0.001), compared to the *L* group (570 ± 131 ml and 481.6 ± 123.28 ml), respectively (Table 2).

Hemodynamics: no significant differences were recorded among groups in the mean MAP at other time points, or in the mean heart rate or SPO_2_ at any studied time point (data not displayed). The HR and NIBP were stable during the whole process.

### 3.2. In the Postoperative Period

The pain was evaluated using the CHEOPS score to assess the necessity for rescue analgesia. The CHEOPS scores were significantly lesser in (the LD group). We found that during the initial 120 min, i.e., from baseline to 120 min, a *P* value > 0.050 was insignificant. The difference in CHEOPS scores between the two groups is significant at 6^th^ and 10^th^ postoperatively with *P*=0.007 and 0.000, respectively. LD group had lesser scores of CHEOPS at almost all-time intervals (Table 3).

### 3.3. Postoperative Sedation

It was evaluated using the Ramsay sedation score in the first 240 minutes after surgery, reduced with time in both groups. The mean sedation values in the LD group were higher than those in the *L* group at almost all points in time but there was no significant difference (Table 3).

### 3.4. Side Effects

Of the 90 patients, 11 vomited, 6 vomited one time (2 in group *L* and 4 in group LD), and 5 suffered from excessive secretion (3 in group *L* and 2 in group LD) with no statistical differences between the groups. No active intervention was performed. Laryngospasm was significantly higher in (the *L* group). The occurrence of laryngospasm was observed in five children in (the *L* group). No active interference was done, and it was self-limiting. However, no child had laryngospasm in the LD group (Table 4).

No patient reported prolonged additional oxygen demand, respiratory depression, tachycardia, arrhythmia, hypo or hypertension, or tonsil bed hemorrhage.

One week of follow-up, no postoperative bleeding was reported, primary or secondary infection with a tonsillar bed that healed optimally one week after the operation without any complaints from the patient.

### 3.5. Patients' Satisfaction

Assessed with ‘‘Likert scale” was adequate (very satisfied, satisfied, and neutral) in almost 97.7% of the LD group as equated to 86.6% in the *L* group, *P* < 0.050 (Table 4).

## 4. Discussion

The most important finding of this study was that an intraoperatively administered dose of 1 *μ*g/kg of a peritonsillar combination of levobupivacaine and dexmedetomidine (LD group) before the start of the adenotonsillectomy operation improved postoperative analgesia, increased the time to the first analgesic call, and decrease the must for postoperative analgesia. In addition, equaled to levobupivacaine alone, this mixture resulted in higher total oral intake and increased family satisfaction on the day after surgery without raising the risk of problems.

In children, determining the severity of pain is critical for treatment and follow-up. It is difficult to adequately quantify pain in children because their cognitive and verbal communication skills are lacking. As a result, employing established criteria to monitor findings should lead to accurate pain diagnosis and treatment [17]. In this study, we attempted to offer a precise evaluation using CHEOPS.

According to Jebeles et al., post-tonsillectomy pain is assisted by the harmful motivation of C-fiber imports in the peritonsillar area [18] and it is induced by nerve inflammation and frustration, as well as spasms of the bare pharyngeal muscles. After surgery, the pain does not disappear entirely until the mucous membrane covers the muscles [19].

They were adding dexmedetomidine to ropivacaine for local anesthesia infiltration enhanced analgesic efficiency, and increased the degree of pain relief after tonsillectomy and adenoidectomy, according to Hao et al. [20] which is similar to our findings. Others found that managing postoperative pain following adenotonsillectomy infiltration with local anesthetics reduced morbidity and improved satisfaction [21]. However, their limited assessment only lasted for the first 24 hours, and they did not continue to track patients or repair surgical techniques and instruments, which are critical factors in postoperative pain assessment, as this study did. In the present study, the same procedure (total bed dissection ± curettage adenoidectomy) was used in all patients to avoid unfair results.

The *α*_2_-adrenergic agonist's analgesic properties might be facilitated over supraspinal, spinal, and peripheral actions [22]. The decline in analgesic requests in this current study was in agreement with earlier studies in adults [23] and pediatrics [24] which settled that IV dexmedetomidine intraoperatively significantly reduced the postoperative need for opioid analgesics. The alteration in this study is the usage of IV paracetamol as rescue analgesia because our institute protocols favor nonopioid analgesia for post-tonsillectomy pain.

In this trial, laryngospasm was significantly greater with levobupivacaine alone, showing the potential advantage of dexmedetomidine in upper airway surgery when paired with local anesthesia. The smooth muscle relaxation induced by local dexmedetomidine infiltrations supports this. The cholinergic EFS-induced contractions and acetylcholine release were reduced by the 2-adrenoceptor agonist dexmedetomidine, indicating the existence of inhibitory 2-adrenoceptors on the prejunctional side of the postganglionic junction between cholinergic neurons and smooth muscles. Exogenous acetylcholine-induced contraction and C-fiber-mediated contraction were both reduced by dexmedetomidine, indicating a direct influence on airway smooth muscle and an underlying mechanism for cough suppression, respectively [25].

The previous study conducted by El-Anwar et al. also found that laryngospasm was higher in the levobupivacaine group [26].

The absence of systemic effects in this study's peritonsillar injections of levobupivacaine and dexmedetomidine suggests that a direct local effect is possible. However, we could not rule out a central analgesic impact due to systemic absorption, the reason for the thickening of the blood vessels in this location. We could not determine plasma levels of levobupivacaine and dexmedetomidine to compare with clinical outcomes that may have established local effects because we did so incorrectly. More research is needed to discover the appropriate amount of analgesics for levobupivacaine and dexmedetomidine in children and to explain local side effects.

The lack of a preoperative gag reflex measurement is one of the study's limitations. It is possible that the lack of a gag reflex after surgery is due to a lack of one before surgery, which can happen in some people. However, assessing swallowing difficulties and parental satisfaction was subjective and could be influenced by other factors, such as the patient's effort.

In future studies, we need to determine optimal dosage requests for other pediatric subpopulations.

## 5. In Conclusion

In children, peritonsillar infiltration of levobupivacaine and dexmedetomidine extremely improved postoperative pain after adenotonsillectomy. The topically applied levobupivacaine and dexmedetomidine were concomitant with no systemic side effects, a higher net oral intake on the first day after surgery, and a better level of family satisfaction.

## Figures and Tables

**Table 1:** Demographic data, surgery type, duration of anesthesia, and extubation time.

		Group L	Group LD	*P*-value
Age (year)		5.2 ± 1.3	5.1 ± 1.3	0.704
Sex M/F		27/18	30/15	0.512
ASA I/II		38/7	39/8	0.764
Weight (kg)		20.32 ± 4.98	19.65 ± 4.41	0.502
Height (cm)		105.2 ± 11.06	105.9 ± 9.75	0.755
Type of surgery:				
Tonsillectomy		24	30	0.197
Adenotonsillectomy		21	15	
Duration of anesthesia		41.8 ± 3.1	42.8 ± 3.8	0.155
Duration of surgery		36.1 ± 3.8	35.8 ± 3.3	0.678
Extubation time		5.1 ± 0.8	6.2 ± 0.7	0.000^*∗∗*^
Recovery time		10.2 ± 1.67	10.8 ± 1.37	0.066

Data presented as mean ± SD and number. Group L: levobupivacaine and Group LD: levobupivacaine plus DEX. Independent sample *t*-test and Chi-square test. ^*∗*^A statistically significant difference (*P* < 0.050). ^*∗∗*^A statistically significant difference (*P* < 0.001).

**Table 2:** Time to first request, total consumption of postoperative IV paracetamol rescue analgesia, total fluid, and semifluid intake.

	Group L (*n* = 45)	Group LD (*n* = 45)	*P*-value
1st rescue analgesia	551.51 ± 146.16	644.31 ± 112.89	0.001^*∗∗*^
No doses in 24 hr			
One dose	32 (71.1%)	40 (88.9%)	0.035^*∗*^
Two doses	13 (28.9%)	5 (11.1%)	
Total consumption of postoperative IV paracetamol	394.89 ± 183.71	321.89 ± 93.25	0.020^*∗*^
Total fluid intake in 24 hours	570 ± 131	725.33 ± 95.12	0.000^*∗∗*^
Total oral semisolid intake in 24 hours	481.6 ± 123.28	630.9 ± 139.39	0.000^*∗∗*^

Data presented as mean ± SD and number (%). Group L: levobupivacaine and Group LD: levobupivacaine plus DEX. Independent sample *t*-test and Chi-square test. ^*∗*^A statistically significant difference (*P* < 0.050). ^*∗∗*^A statistically significant difference (*P* < 0.001).

**Table 3:** Postoperative CHEOPS and ramsay sedation score.

	Group L	Group LD	*P*-value
CHEOPS score			
CHEOPS 0 min	4 (3–6)	4 (3–6)	0.914
CHEOPS 30 min	4 (3–6)	5 (3–6)	0.203
CHEOPS 60 min	5 (3–6)	5 (3–6)	0.806
CHEOPS 90 min	5 (3–6)	4 (3–6)	0.078
CHEOPS 2 hr	5 (3–6)	5 (3–6)	0.171
CHEOPS 6 hr	6 (3–8)	5 (3–7)	0.007^*∗*^
CHEOPS 10 hr	6 (3–8)	5 (3–7)	0.000^*∗*^
CHEOPS 12 hr	7 (4–8)	7 (4–8)	0.481
CHEOPS 24 hr	5 (3–8)	5 (3–6)	0.394

Ramsay sedation score			
RSS 0 min pacu	4 (2–5)	4 (2–5)	0.426
RSS 15 min	3 (1–5)	3 (2–4)	0.527
RSS 30 min	2 (1–4)	2 (1–3)	0.432
RSS 60 min	1 (1–3)	2 (1–3)	0.156
RSS 120 min	1 (1–3)	1 (1–3)	0.541
RSS 180 min	1 (1–2)	1 (1–2)	1.000
RSS 240 min	1 (1–2)	1 (1–2)	0.559

Data presented number (%). Group L: levobupivacaine and Group LD: levobupivacaine plus DEX. The Mann–Whitney *U* test. ^*∗*^A statistically significant difference (*P* < 0.050). ^*∗∗*^A statistically significant difference (*P* < 0.001).

**Table 4:** Postoperative side effects and likert score.

	Group L (*n* = 45)	Group LD (*n* = 45)	*P*-value
POV	5 (11.1%)	6 (13.3%)	0.748
Laryngospasm	5 (11.1%)	0	0.021^*∗*^
Abdominal pain	3 (6.7%)	1 (2.2%)	0.306
Hypotension	2 (4.4%)	4 (8.9%)	0.398
Bradycardia	2 (4.4%)	1 (2.2%)	0.557

Satisfaction score			
Strongly dissatisfied	2 (4.4%)	0	0.016^*∗*^
Dissatisfied	4 (8.9%)	1 (2.2%)	
Neutral	10 (22.2%)	3 (6.7%)	
Satisfied	17 (37.8%)	16 (35.6%)	
Strongly satisfied	12 (26.7%)	25 (55.6%)	

Data presented number (%). Group L: levobupivacaine and Group LD: levobupivacaine plus DEX. Chi-square test. ^*∗*^A statistically significant difference (*P* < 0.050).^*∗∗*^A statistically significant difference (*P* < 0.001). POV (Postoperative vomiting).

## Data Availability

The data used to support the findings of this study are available from the corresponding author upon request.
